# Flow and Satisfaction With Life in Elite Musicians and Top Athletes

**DOI:** 10.3389/fpsyg.2019.00698

**Published:** 2019-03-29

**Authors:** Katarina Habe, Michele Biasutti, Tanja Kajtna

**Affiliations:** ^1^Academy of Music, University of Ljubljana, Ljubljana, Slovenia; ^2^Department FISPPA, University of Padova, Padova, Italy; ^3^Faculty of Sport, University of Ljubljana, Ljubljana, Slovenia

**Keywords:** flow, satisfaction with life, expert performance, top athletes, elite musicians

## Abstract

Although flow has been studied extensively in music and sport, there is a lack of research comparing these two domains. With the aim of filling this gap, elite musicians and top athletes in Slovenia were contrasted in the current study. Differences for flow and satisfaction with life between elite musicians and top athletes were explored. Individual versus group performance setting and gender differences were considered. 452 participants; 114 elite Slovenian musicians (mean age 23.46 years) and 338 top Slovenian athletes (mean age 22.40 years) answered questions about flow and satisfaction with life measures. The results show differences between elite musicians and top athletes in four flow dimensions: transformation of time and autotelic experience were higher in musicians while clear goals and unambiguous feedback were higher in athletes. However, differences in global flow were not confirmed. Elite musicians and top athletes experienced flow more often in group than in individual performance settings and surprisingly it was experienced more in male than in female top performers. Satisfaction with life has a positive correlation with all nine dimensions of flow, but only challenge-skill balance was a significant predictor for satisfaction with life.

## Introduction

There is a growing interest in research into flow in music and sport. These two fields have several aspects in common, such as that both music and sport are performative activities in which the performers have to give their best. The concert is the typical situation in which musicians have to produce a peak performance, and as contests and matches are also where athletes have to produce performance excellence. There are several studies that have associated flow with peak performance (O’Neill, 1999; MacDonald et al., 2006; Baker and MacDonald, 2013; Marin and Bhattacharya, 2013). Flow was extensively studied in top athletes (Lindsay et al., 2005; Nicholls et al., 2005; Sugiyama and Inomata, 2005; Aherne et al., 2011; Pain et al., 2011; Pates et al., 2012; Briegel-Jones et al., 2013; Pates and Cowen, 2013; Hodge et al., 2014; Koehn et al., 2014; Norsworthy et al., 2017; for review, see Swann et al., 2012), but there is a scarcity of data exploring flow in elite musicians (Sinnamon et al., 2012). Further, flow in music and sport has typically been considered separately without comparing these two domains.

There may also be other relevant points of comparison for musicians and athletes, such as satisfaction with life. Satisfaction with life is connected to the stressful life that musicians and athletes lead, but few studies have investigated the relationship between flow and satisfaction with life in top/elite performers. Although previous studies have concentrated mainly on the measurement of affective well-being – considering flow as an emotional phenomenon – the measurements of life satisfaction could complement traditional affective measurements which has proven useful in predicting reactions to stressful events (Huebner and Dew, 1996; Bradley and Corwyn, 2004).

Trying to fill this gap, the current study contrasted elite musicians and top athletes for flow and satisfaction with life. Individual versus group performance settings and gender differences were considered. The literature review covers several issues, including defining performance excellence, similarities and differences between sport and music performance, flow and music, flow and sports, and flow and satisfaction with life.

### Defining Performance Excellence

There are many synonyms for performance excellence, such as peak performance, expert performance, elite performance, top performance, optimal performance and performance expertise (Berkopec, 2016). Peak performance is defined as: “quantitatively and/or qualitatively absolutely outstanding action results achieved under regular conditions” (Nitsch and Hackfort, 2016, p. 15). In this concept the following three features are emphasized: “peak performance implies acting at one’s own limits, it requires full commitment to the expert activity and often neglecting all other life areas and it brings publicity to the performer” (Nitsch and Hackfort, 2016, p. 15). There are several differences in music and sport when defining excellence. The criteria are often far more subjective in the music domain than in sport. In sport scores and timings are all that counts, and the main criteria of success involve quantity. Conversely, musical excellence is a result of a subtle unreachable interplay between musical piece, performer/s, performance setting and audience (Waddell et al., 2018). When talking about performance excellence in the case of music and sport, a performance should be referred to as work rather than as leisure (Juniu et al., 1996; Delle Fave et al., 2011c; Delle Fave and Zager Kocjan, 2016).

### Similarities and Differences Between Sport and Music Performance

Sport and music performances share many similarities, such as requirements for prolonged training, endurance, perfectionism, high self-regulation, strategic decisions, emotional expressivity, and social skills. Music facilitates several elements of sport performance such as arousal regulation, synchronization, acquisition of motor skills (Pates et al., 2003).

With regard to the differences, music training often starts earlier than sports training involves fine-motor skills, and takes place in extremely competitive environments, that often cause anxiety even in top performers (Biasutti and Concina, 2014). Anxiety in top performers is not necessarily due to the competitive environment, but also because the evaluation of what makes a good musical performance is subjective, thus introducing a larger degree of uncertainty than in sports. However, also sport performances take place in particularly competitive settings which could be subjected to external factors and evaluations. In sports such as figure skating, dancing, artistic gymnastics the performance is assessed by judges and this could cause additional stress for the characteristics of the evaluation process. In addition, emotional states and expressivity are frequently part of success, because for athletes it is important to express their feelings as opposed to holding them down and thus increasing tension, which might disrupt performance (Kajtna and Jeromen, 2013).

Musical and sport performances are frequently linked to emotional states, such as flow (Martin, 2008; Altenmüller and Ioannou, 2016). One common aspect of music and sport performance is that both have a strong potential to elicit flow. Csikszentmihalyi (1990, 1993) found that artists and athletes seemed more likely to experience flow, especially during their work. Csikszentmihalyi (1975, p. 36) defined flow as “the holistic sensation that people feel when they act with total involvement.” Flow refers to a state of mind which brings together cognitive, physiological and affective aspects and corresponds to an optimal psychophysical state (Biasutti, 2017). In music and sport total immersion with, absorption in the activity, high internal enjoyment and intrinsic motivation are frequently observed. Entering a flow state involves developing a balance between the perceived abilities of a person and their perceived opportunities (Nakamura and Csikszentmihalyi, 2002), and balancing challenges and skills is the golden rule of flow (Jackson and Csikszentmihalyi, 1999). The experience of flow is characterized by the following nine dimensions: a challenge-skill balance, merging of action and awareness, clear goals, unambiguous feedback, total concentration, sense of control, loss of self-consciousness, transformation of time, and autotelic experience (Csikszentmihalyi, 1990).

### Flow and Music

Since music is noted as one of the activities which induces flow most often (Lowis, 2002), it is not surprising that in recent years, many studies have been conducted on flow in the musical domain (O’Neill, 1999; Custodero, 2002, 2005; MacDonald et al., 2006; Smolej Fritz and Avsec, 2007; Biasutti and Frezza, 2009; de Manzano et al., 2010; Diaz, 2013; Fullagar et al., 2013; Wrigley and Emmerson, 2013; Biasutti, 2015; Chirico et al., 2015; Hart and Di Blasi, 2015). Music in its origins is a kind of flow of structured sounds in time and space with emotional and cognitive content. When we are immersed in music with full concentration – with the whole of our being – we have the opportunity to access the state of flow. According to Croom (2012, 2015) the analysis of flow in musical contexts is a rapidly-developing field. Chirico et al. (2015) reported that when studying flow in music, it is necessary to concentrate on different music activities, such as musical composition (Byrne et al., 2003; John, 2006; MacDonald et al., 2006; Hart and Di Blasi, 2015), musical listening (Diaz, 2013) and musical performance (O’Neill, 1999; Sawyer, 2006, 2007; Smolej Fritz and Avsec, 2007; Kirchner, 2011). Most studies of flow in the musical domain have focused on musical performance.

### Flow and Sports

There are even more studies exploring flow in the sports domain than in the music domain (Csikszentmihalyi et al., 1977; Massimini and Carli, 1988; Jackson, 1996; Jackson and Marsh, 1996; Jackson et al., 1998; Jackson and Csikszentmihalyi, 1999; Bakker et al., 2011; Muzio et al., 2012; Swann et al., 2012). In sports, the majority of studies have been conducted with top athletes and only a few with recreational athletes (Jackson, 1996; Jackson and Marsh, 1996; Jackson et al., 1998; Bakker et al., 2011). Many of these studies used qualitative methodology as a source of in-depth data for confirming the nine dimensional constructs of flow, proposed by Jackson and Csikszentmihalyi (1999). Jackson (1992) in a qualitative study of figure skaters concluded that flow is defined by high levels of challenges and skills, attention and a clear, perfect focus. Similar conclusions were drawn with Japanese University athletes coming from different areas of sports such as swimming, athletics, and figure skating (Sugiyama and Inomata, 2005), and with elite swimmers (Bernier et al., 2009).

### Differences in Experiencing Flow With Regard to Gender and Individual/Group Performance Settings

Considering gender differences in experiencing flow Csikszentmihalyi (1990, p. 4) describes, that “Flow is reported in essentially the same words by men and women.*”* This descriptive finding suggests that flow is broadly reported beyond gender (Bonaiuto et al., 2016). Altought most of the studies show no differences in experiencing flow between men and women (Russell, 2001; Kee and Wang, 2008; Moreno et al., 2008; Bonaiuto et al., 2016), some studies suggest, that flow is experienced more frequently by female than by male (Gnezda, 2016; Habe and Berkopec, 2016; Habe and Tement, 2016). For example a higher sense of control (flow dimension) in boy athletes compared to girl athletes between 12 and 16 years old was reported by Moreno et al. (2008).

In the current study gender differences in flow are expected because women tend to experience positive emotional states in a more intensive and vivid way than men do (Fujita et al., 1991).

In addition, differences in experiencing flow according to performance setting (individual/group) are expected. Sawyer (2003) defined group flow as a collective state that occurs when a group is performing at the peak of its abilities. The conditions that encourage individual flow could also influence group flow (Sawyer, 2006). Duncan and West (2018) offer a simplified model of group flow, consisting of three dimensions: vision, ownership & contribution, communication. When groups cooperate to agree on goals and patterns, social flow, commonly known as group cohesion, is more likely to occur than individual flow (Walker, 2010). Interestingly previous studies did not show significant differences in experiencing flow between individual and team sport performance (Fossmo, 2006; Elbe et al., 2010). Conversely, in music performance flow is experienced more frequently in group performance than in individual performance, because the level of performance anxiety is lower (Bloom and Skutnick-Henley, 2005; Berkopec, 2016).

### Flow and Satisfaction With Life

Flow can be considered one of the indicators of well-being. Experiencing flow brings performers a sense of accomplishment and internal success. In top performers being subjected to flow conveys not only emotional fulfillment, but also a general feeling of satisfaction with life. Satisfaction with life could be considered a cognitive and global evaluation of the quality of one’s life as a whole (Pavot and Diener, 1993).

Top/elite performers face stressful and challenging events on an almost everyday basis. Satisfaction with life, could be considered a cognitive indicator of subjective well-being. Flow is also frequently experienced during performance, which is a core component of a professional career. Flow can be viewed as a facilitator for perceiving internal success in performance, which can reflect satisfaction with life. Flow can also be perceived as positive stress (eustress), which is defined as a stress that results in positive outcomes (Selye, 1975). Eustress is usually the result of more manageable levels of stress (Le Fevre et al., 2003). The idea of eustress as “good stress” relates to the Yerkes-Dodson Law, which states that increasing arousal is beneficial to performance until some optimum level is reached (O’Sullivan, 2011). Perceived positive stress is positively related to life satisfaction (Abolghasemi and Taklavi Varaniyab, 2010). If we consider flow as a kind of positive stress, then it requires extensive activation at all levels of human functioning. To reach peak performance, a cognitive component of flow seems to play a crucial role in gaining personal well-being (Bloom and Skutnick-Henley, 2005). Flow has received attention as being among the indicators of an individual’s well-being (Csikszentmihalyi, 1975; Bassi and Delle Fave, 2012).

One of the basic functions of music is to promote well-being (Thorgaard et al., 2004; Hays and Minichiello, 2005), which also happens in recreational sports (Arent et al., 2000; Peluso and de Andrade, 2005; Ströhle, 2009; Häkkinen et al., 2010). However there is a difference regarding the expertise of performers. As opposed to amateur musicians or recreational athletes, many performing experts often report on the negative aspects of their elite performing career, such as anxiety, depression and stress (Kenny et al., 2003; Murko Feguš, 2016).

Well-being can be viewed from a hedonic or eudemonic perspective. The hedonic paradigm is represented by subjective well-being, divided into cognitive (satisfaction with life) and emotional (positive/negative emotionality) components (Diener et al., 1985). Conversely, the eudemonic paradigm is represented by psychological well-being which comprises self-acceptance, personal growth, purpose in life, environmental mastery, autonomy, and positive relations with others (Ryff, 1989).

To our knowledge there is little research that has explored well-being in professional athletes. Previous studies on flow and well-being reveal that experiencing more intense flow correlates positively with hedonic and eudemonic well-being (Smolej Fritz and Avsec, 2007; Delle Fave et al., 2011a; Moneta, 2012; Bassi et al., 2014; Baker et al., 2015).

Other studies about the relationship between flow and satisfaction with life in musicians show that satisfaction with life is weakly positively associated with flow, while having clear goals supports a sense of satisfaction with life (Chirico et al., 2015). Clear goals are important predictors of satisfaction with life (Smolej Fritz and Avsec, 2007), and two dimensions of flow, balance between challenge and skills and autotelic experience, correlated positively with satisfaction with life (Sedlár, 2014). Smolej Fritz and Avsec (2007) concluded that the experience of flow in musicians is more related to emotional than cognitive aspects of subjective well-being. In accordance with previous research findings on flow, we hypothesized that:

• **H1:** There would be differences in flow between elite musicians and top athletes.
• **H2:** There would be differences in flow regarding group versus individual performance settings.
• **H3:** There would be gender differences in flow with female top/elite performers experiencing flow more often than male.
• **H4:** Flow in top/elite performers would positively correlate with satisfaction with life.

## Materials and Methods

### Participants

Four hundred and fifty two elite musicians and top athletes participated in the study. Elite musicians are expert active performers that regularly perform nationally and internationally as soloists or as members of small chamber groups (duo, trio, quartet, quintet) in eminent solo concerts and festivals and/or have gained renowned rewards (first, second, third prize) in national and/or international competitions. The inclusion criteria for elite musicians is to have a career as a musician and to regularly perform internationally or having gained a prize in national or international competitions. Top athletes are athletes that play individually or in teams at national level. The inclusion criteria for top athletes is registration in the Olympic committee of Slovenia – the Association of Sports Federations. Participant ages ranged between 14 and 58 years, with a mean age of 23.46 years (*SD* = 7.06 years). There were 114 top Slovenian musicians with a mean age of 26.59 years (*SD* = 9.46 years) and 338 top Slovenian athletes with a mean age of 22.40 years (*SD* = 5.68 years). Musicians included wind instrument players (27.2%), pianists and accordion players (24.6%), singers (21.1%) and string instrument players (22.6%). Athletes were 63.0% individual sports athletes (swimming, track and field athletes, tennis players, martial arts athletes) and 37.0% team sports athletes (handball, football, basketball and volleyball). The difference in age between musicians and athletes was significant [*t* = -4.46; *p*(*t*) = 0.00]. 149 participated in team events (team sports or bands and choirs) and 303 in individual sports or mainly undertook solo performances. The differences in age between participants who participated in team events and those who perform individually were not significant [*p*(*t*) = 0.68]. 224 participants were male and 224 were female, the differences in age were significant [*t* = 2.43; *p*(*t*) = 0.02]- the male mean age was 24.25 years (*SD* = 7.54 years) and the female was 22.65 years old on average (*SD* = 6.45).

This study was carried out in accordance with the recommendations of the Slovenian Psychological Society. In accordance with the Declaration of Helsinki consent obtained from all adult participants and from the parents of all 6 participants under the age of 16 was both written and informed. Research was approved by the Ethical committee of the Faculty of Arts at the University of Maribor.

### Instruments

#### Dispositional Flow Scale – 2 (DFS-2; Jackson and Eklund, 2002)

DFS-2 is a self-descriptive instrument, based on the following nine dimensions of flow theory (Csikszentmihalyi, 1990): challenge-skill balance, merging of action and awareness, clear goals, unambiguous feedback, total concentration, sense of control, loss of self-consciousness, transformation of time, autotelic experience. It includes 36 items, 4 items per dimension. The items refer to athletic and physical dimensions, during which people experience intense positive emotions. Each item is evaluated on a five level Likert scale, where 1 means “never” and 5 “always.” The respondents are asked to evaluate how often they experience the sensation described for each item. A global flow score was computed, considering the sum of all items. The reliability of the scale was high, with Cronbach alpha coefficients ranging between 0.78 and 0.86 (Phillips, 2005).

#### Satisfaction With Life Scale (SWLS; Diener et al., 1985)

SWLS is a scale composed of five items, which measure the overall self-estimation of quality of life. Participants evaluate their level of agreement with each item on a seven level Likert scale, where 1 means “completely disagree” and 7 “completely agree.” The score was obtained by summing up the five items, 35 being the maximum score. The questionnaire has shown high internal consistency and stability over time, ranging up to 4 years (Pavot and Diener, 1993). The initial study had a Cronbach alpha of 0.87 and test–retest reliability of 0.82 (Diener et al., 1985).

### Procedure

The participants were recruited through national sports associations and musical schools and orchestras. The measures were administered individually in face-to–face meetings in small groups, mainly after practice or rehearsal sessions.

## Results and Discussion

The data was processed with the statistical package IBM SPSS 21.0. Differences between athletes and musicians and male and female participants, were computed with Ancova as we controlled the effect of age. Participants, who mainly perform individually, and those who mainly perform in groups were compared using a *t*-test, and correlations between flow dimensions and satisfaction with life was calculated with Pearson’s r. Linear regression was used to predict satisfaction with life on the basis of flow experience.

The first analysis was of reliability on all the measures for assessing the quality of the data collected. The results have shown high reliability: Cronbach’s Alpha was 0.85 for SWLS and from 0.74 to 0.83 for the subscales of DFS-2, while global flow reliability was 0.83.

Regarding the first hypothesis (*There would be differences in some dimensions of flow between elite musicians and top athletes*) the comparison of elite musicians and top athletes in flow dimensions and satisfaction with life shows that there are significant differences in four dimensions of flow (see Table 1).

**Table 1:** Comparison of athletes and musicians in flow dimensions and satisfaction with life.

Measures	Athletes		Musicians			
	*M*	*SD*	*M*	*SD*	*F*	*p*
Challenge-skill balance	15.74	2.25	15.64	2.12	0.41	0.68
Merging of action and awareness	14.27	2.83	14.55	2.32	-1.06	0.29
Clear goals	17.24	2.24	16.61	2.39	2.56	0.01
Unambiguous feedback	15.89	2.45	14.72	2.64	4.33	0.00
Total concentration	15.86	2.38	15.63	2.32	0.87	0.38
Sense of control	15.12	2.45	15.13	2.31	-0.04	0.97
Loss of self-consciousness	13.75	3.70	13.66	3.86	0.22	0.83
Transformation of time	14.08	3.82	16.03	2.91	-5.68	0.00
Autotelic experience	16.20	2.64	16.84	2.20	-2.56	0.01
Global flow score	138.14	16.63	138.81	14.81	-0.38	0.70
Satisfaction with life	24.45	5.57	25.82	5.20	-2.13	0.03

*M, mean; SD, standard deviation; p, statistical significance of parameter F. The measures highlighted in gray had significant differences between athletes and musicians*.

These differences have different trends: when athletes experience flow they have a clear idea of what their goals are and they have clear feedback, which tells them that they are on the way to achieving them. Conversely, elite musicians experience more transformation of time, which can either go faster or slower and they have a more autotelic experience of their performance than the top athletes. These findings are consistent with the different characteristics of the two disciplines: it is probably easier to set goals in sport where you can easily control their achievement. For example when you are running 100 meters you can tell using a chronometer whether you have run below a specific time, and can develop feedback mechanisms for monitoring the achievement of goals. Conversely, music is an artistic discipline subject to individual interpretation, and is more difficult than in sports to set clear and shared goals and to control them. Elite musicians demonstrated a deeper level of transformation of time than top athletes, which is a characteristic connected to the nature of music. Music is developing over time and can induce trance and altered states of consciousness (this issue will be discussed in more depth later). Our results about the differences in transformation of time between elite musicians and top athletes are not in line with previous findings by Thomson and Jaque (2016), that showed no differences between talented dancers, singers and athletes. A possible reason could be due to the different population of participants included in the current and in the Thomson and Jaque’s (2016) studies. The difference in transformation of time between musicians and athletes maybe emerges only at a certain level of expert skills and by the most outstanding performers. It is relevant to note that the autotelic experience is more pronounced in elite musicians than in top athletes, and represents a pleasant experience which is intrinsically rewarded, since the gratification is directly connected with the activity performed. Satisfaction with life seems to be higher in elite musicians when compared to top athletes. In this case the level of significance is not high and this data needs to be confirmed in subsequent research.

Regarding the second hypothesis (*There would be differences in flow regarding group versus individual performance setting*), when we compared performers in team settings (athletes who participate in team sports and musicians who perform mainly in choirs or ensembles) to those who mainly perform individually (individual sports and solo performers in music) we found many differences, with higher scores for team performers.

Team performers have a better balance between challenge and skill, their awareness merges with what they are doing more frequently, they have clearer goals and more frequently experience total concentration than individual performers. They also have more sense of control, experience more transformation of time, have a more autotelic experience and the global flow score is higher in team performers (see Table 2). Conversely, the differences in satisfaction with life between team and individual performers are not statistically significant.

**Table 2:** Comparison of team and individual performers in flow dimensions and satisfaction with life.

	Team		Individual			
Measures	*M*	*SD*	*M*	*SD*	*t*	*p*
Challenge-skill balance	16.13	2.05	15.51	2.27	2.84	0.00
Merging of action and awareness	14.91	2.38	14.06	2.82	3.32	0.00
Clear goals	17.42	1.98	16.91	2.42	2.21	0.03
Unambiguous feedback	15.91	2.50	15.44	2.56	1.86	0.06
Total concentration	16.17	2.23	15.62	2.41	2.40	0.02
Sense of control	15.46	2.26	14.96	2.48	2.06	0.04
Loss of self-consciousness	13.73	3.53	13.72	3.84	0.03	0.97
Transformation of time	15.28	3.34	14.22	3.83	2.86	0.00
Autotelic experience	16.98	2.26	16.06	2.62	3.85	0.00
Global flow score	141.98	14.65	136.50	16.61	3.42	0.00
Satisfaction with life	25.42	4.75	24.82	5.68	0.82	0.41

*M, mean; SD, standard deviation; p, statistical significance of parameter t. The measures highlighted in gray had significant differences between teams and individuals*.

Table 3 shows several differences in experiencing flow between athletes and musicians in team and individual performances. The previously described differences correspond fully to the subsample of athletes, while in musicians only the transformation of time corresponds to those differences between individual and team performers. The differences in experiencing flow in athletes according to our results are not consistent with previous studies, which showed no significant differences in experiencing flow between individual and team sport performance (Fossmo, 2006; Elbe et al., 2010). The differences in our research may be a consequence of the selected population – top athletes – which have very developed performance skills. Conversely, previous studies confirmed the differences in musicians experiencing flow, showing that flow is experienced more frequently in group performing than in individual performing (Bloom and Skutnick-Henley, 2005; Berkopec, 2016). There were significant differences only for the transformation of time in elite musicians in our study.

**Table 3:** Comparison of team and individual performers in musicians and athletes in flow dimensions and satisfaction with life.

Measures	Athletes					Musicians				
	Team		Individual			Team		Individual		
	*M*	*SD*	*M*	*SD*	*t*	*M*	*SD*	*M*	*SD*	*t*
Challenge-skill balance	16.26	2.08	15.44	2.30	3.28**	15.50	1.79	15.68	2.20	-0.36
Merging of action and awareness	14.92	2.38	13.89	3.01	3.49**	14.83	2.44	14.48	2.29	0.67
Clear goals	17.56	1.99	17.05	2.36	2.04*	16.67	1.76	16.59	2.54	0.17
Unambiguous feedback	16.13	2.44	15.75	2.45	1.37	14.79	2.57	14.70	2.68	0.15
Total concentration	16.26	2.22	15.62	2.44	2.45*	15.71	2.24	15.61	2.35	0.18
Sense of control	15.47	2.30	14.92	2.52	2.02*	15.38	2.08	15.07	2.37	0.58
Loss of self-consciousness	13.83	3.52	13.69	3.81	0.33	13.21	3.56	13.78	3.94	-0.64
Transformation of time	14.91	3.46	13.59	3.94	3.11**	17.17	1.69	15.72	3.10	3.05**
Autotelic experience	16.90	2.31	15.79	2.73	4.00**	17.38	2.04	16.70	2.23	1.34
Global flow score	142.24	14.82	135.73	17.19	3.53**	140.63	13.95	138.32	15.07	0.68
Satisfaction with life	24.43	5.09	24.45	5.75	-0.03	27.46	3.19	25.39	5.54	2.37*

*M, mean; SD, standard deviation; ^∗^t significant at 0.05 level; ^∗∗^t significant at 0.00 level. The differences that were significant are highlighted in gray*.

Transformation of time is one phenomenon that characterizes music. A different perception of time could be induced when people are deeply involved during listening music. Also performing music or singing in a group enhances the transformation of time. In ancient civilizations group music making was one of the forms of accessing spiritual dimensions (Dewhurst-Maddock, 1999) and a different perception of time was induced. For musicians, time perception is the core of their flow experience and happens when all the levels of existence (body, emotions, mind, and spirit) merge into one whole during the performance. Elite musicians had a different trend than top athletes for satisfaction with life, which was higher for musicians who mainly perform in group settings.

When we compared male and female participants regarding the third hypothesis (*There would be gender differences in flow with female top/elite performers experiencing flow more often than male*), we found several differences in flow dimensions but no difference in satisfaction with life (as reported in Table 4). Males experienced flow more frequently, both in general, as the global flow score shows us, as well as in individual dimensions of flow. Males experienced a higher balance between the challenge of the situation and their skills, feeling that they had clearer feedback about the situation and more control when they are performing than did females. Males also experienced total concentration and loss of self-consciousness more frequently. These results are rather surprising because in the general population, dimensions of well-being and flow are usually more pronounced in females than in males. Gender differences can be partially explained by the fact that women tend to experience positive emotional states in a more intensive and vivid way than do men (Fujita et al., 1991).

**Table 4:** Comparison of male and female participants in flow dimensions and satisfaction with life.

Measures	Male		Female			
	*M*	*SD*	*M*	*SD*	*F*	*p*
Challenge-skill balance	15.96	2.11	15.46	2.30	2.39	0.02
Merging of action and awareness	14.46	2.71	14.22	2.71	0.95	0.34
Clear goals	17.28	2.10	16.87	2.46	1.91	0.06
Unambiguous feedback	15.97	2.41	15.21	2.63	3.22	0.00
Total concentration	16.08	2.24	15.51	2.46	2.60	0.01
Sense of control	15.46	2.23	14.78	2.55	3.06	0.00
Loss of self-consciousness	14.44	3.44	13.00	3.89	4.18	0.00
Transformation of time	14.46	3.83	14.68	3.59	-0.61	0.54
Autotelic experience	16.58	2.37	16.14	2.69	1.86	0.06
Global flow score	140.71	14.50	135.86	17.41	3.21	0.00
Satisfaction with life	25.22	5.46	24.75	5.48	0.75	0.46

*M, mean; SD, standard deviation; p, statistical significance of parameter F. The measures highlighted in gray had significant differences between males and females*.

As in the previous set of comparisons, we found that when we compared male and female musicians and athletes separately, the differences shown in the entire sample of participants correspond to the differences in athletes, while there were no differences between male and female musicians in the experience of flow (see Table 5). These findings are coherent with the assumption that musicians are more androgynous than the general population (Kemp, 2000). According to Bem’s theory of psychological androgyny (1974, in Kemp, 2000), gender roles may involve a four-fold typology; masculine, feminine, androgynous and undifferentiated. In general, musicians appear to be able to extricate themselves from the major influences of the gender stereotypes. They may minimize differences in gender-related traits (Kemp, 2000). However, in the current study musicians and athletes were compared rather than musicians and general population. These findings could also be explained because the physical differences between male and female athletes have a larger effect in sport than in music performers.

**Table 5:** Comparison of male and female musicians and athletes in flow dimensions and satisfaction with life.

Measures	Athletes					Musicians				
	Male		Female			Male		Female		
	*M*	*SD*	*M*	*SD*	*t*	*M*	*SD*	*M*	*SD*	*t*
Challenge-skill balance	16.10	2.10	15.34	2.35	3.14**	15.47	2.08	15.78	2.15	-0.77
Merging of action and awareness	14.48	2.77	14.04	2.89	1.44	14.39	2.55	14.68	2.13	-0.66
Clear goals	17.43	2.07	17.02	2.40	1.66	16.76	2.16	16.48	2.58	0.64
Unambiguous feedback	16.28	2.31	15.46	2.53	3.11**	14.90	2.48	14.57	2.77	0.66
Total concentration	16.15	2.20	15.53	2.54	2.41*	15.84	2.38	15.46	2.27	0.88
Sense of control	15.46	2.22	14.75	2.65	2.69*	15.47	2.32	14.86	2.28	1.42
Loss of self-consciousness	14.50	3.39	12.91	3.86	4.01**	14.22	3.65	13.21	3.98	1.40
Transformation of time	14.02	3.94	14.14	3.69	-0.29	16.00	2.95	16.05	2.91	-0.09
Autotelic experience	16.41	2.46	15.98	2.80	1.51	17.20	1.94	16.56	2.36	1.56
Global flow score	140.84	14.63	135.17	18.16	3.14**	140.25	14.18	137.63	15.32	0.94
Satisfaction with life	24.74	5.36	24.17	5.77	0.71	26.06	5.57	25.63	4.91	0.43

*M, mean; SD, standard deviation; ^∗^t significant at 0.05 level; ^∗∗^t significant at 0.00 level. Highlighted in gray are the differences that were significant*.

Regarding the fourth hypothesis (*Flow in top/elite performers would positively correlate with satisfaction with life*) upon correlating dimensions of flow to satisfaction with life, we found significant correlations with all sub-dimensions and the global flow score, but they were quite low (see Table 6). Those that were correlated highly enough for serious discussion were the global flow score and the challenge-skill balance. Our results are consistent with the outcomes of previous studies (Smolej Fritz and Avsec, 2007; Chirico et al., 2015). The challenge-skill balance is important for satisfaction with life in top/elite performers, because this is a dimension over which a performer can have the most control. This sense of control could lower a performer’s stress, resulting in higher satisfaction with life.

**Table 6:** Pearson correlation coefficients between flow dimensions and satisfaction with life.

Flow subscales	Satisfaction with life
Challenge-skill balance	0.36^∗∗^
Merging of action and awareness	0.26^∗∗^
Clear goals	0.22^∗∗^
Unambiguous feedback	0.27^∗∗^
Total concentration	0.29^∗∗^
Sense of control	0.28^∗∗^
Loss of self-consciousness	0.23^∗∗^
Transformation of time	0.24^∗∗^
Autotelic experience	0.29^∗∗^
Global flow score	0.41^∗∗^

^∗∗^*Correlation is significant at the 0.01 level (2-tailed)*.

The participants of this study were all individuals who were highly successful in their area and who perform in competitions at the highest level. In the last analysis we verify whether the experience of flow influenced how satisfied they were with their lives. We calculated a linear regression of flow dimensions on satisfaction with life, but in the first step we excluded the effect of gender and the group (athletes and musicians), as we found some differences between those subgroups in the entire group of participants. As the model is statistically significant (see Table 7), we can argue that the experience of flow influences satisfaction with life in high-performing individuals in music or sports. The main effect is due to the perceived equilibrium between the challenge the situation presents and the perceived set of skills that make a believe they are able to face this particular situation.

**Table 7:** Prediction of satisfaction with life on the basis of flow disposition.

	Dimension of flow	*B*	*β*	*T*	*p (t)*	*R*	*R*^2^	*adj R*^2^	*F*	*p(t)*
Step	Constant	23.84		17.97	0.00	0.13	0.02	0.01	2.62	0.08
1	Group	1.40	0.12	2.16	0.03					
	Gender	-0.52	-0.05	-0.83	0.41					
Step	Constant	4.05		1.34	0.18	0.44	0.19	0.16	6.25	0.00
2	Challenge-skill balance	0.54	0.22	2.94	0.00					
	Merging of action and awareness	0.08	0.04	0.63	0.53					
	Clear goals	0.03	0.01	0.21	0.83					
	Unambiguous feedback	0.26	0.12	1.73	0.08					
	Total concentration	0.20	0.09	1.23	0.22					
	Sense of control	-0.13	-0.06	-0.71	0.48					
	Loss of self-consciousness	0.15	0.10	1.61	0.11					
	Transformation of time	0.17	0.11	1.74	0.08					
	Autotelic experience	-0.05	-0.03	-0.32	0.75					

*B, Unstandardized regression coefficient; β, standardized regression coefficient. The significant dimension is highlighted in gray*.

## Study Strengths, Limitations and Implications

There are several study strengths. First, it is to our knowledge the first study to date that has empirically explored differences in experiencing flow between elite musicians and top athletes. It highlights some important issues of accessing flow in different performance settings (individuals versus groups). It further investigated the relationship between flow and satisfaction with life, which has been rarely explored, finding that one dimension of flow is relevant as a predictor for satisfaction with life.

Our study has some limitations. The principal limitation concerns sampling. The sample might be biased because the more intrinsically motivated performers may have been more likely to take part in the research (Habe and Tement, 2016). The distribution of athletes and musicians differed, because it was harder to get access to elite musicians in comparison to top athletes who are organized through the Olympic Committee. The second limitation is connected to flow assessment and the use of retrospective self-report data which is biased because of the different response styles, personality characteristics, and affective states (Delle Fave et al., 2011b). It would be advisable to use an experience sampling procedure, but according to our experience it is very difficult to collect data from elite musicians and top athletes because top performers are focused on their optimal performance, trying to avoid any kind of distraction.

Future studies should consider applying a longitudinal design to examine stability and change in flow over time (Mäkikangas et al., 2010). It would also be advisable to highlight the relationship between flow and satisfaction with life from the perspective of job demands and job resources in top/elite professional performers. Flow could be associated also with constructs such as self-efficacy or resilience to verify other relevant aspects that could contribute to flow development.

The current study shows that several strategies related to different performance settings are required for accessing the optimal performing state of flow. For regulating satisfaction with life in top/elite performers the most important strategy is balancing skills and performing challenges, where not only physical but also psychological abilities should be taken into account. It would be advisable for more studies in top/elite performance settings to investigate interventions for gaining well-being in professional performers. In addition, participants such as students and trainees could be involved for assessing the differences between experts and novices and the developmental aspects of flow. A research focused on how flow develops could offer several ideas for education and the designing friendly flow environments.

## Data Availability

All datasets generated for this study are included in the manuscript and/or the supplementary files.

## Author Contributions

KH and TK contributed conception and design of the study and organized the database. MB and TK performed the statistical analysis. KH wrote the first draft of the manuscript. KH, MB, and TK wrote sections of the manuscript, contributed to manuscript revision, and read and approved the submitted version.

## Conflict of Interest Statement

The authors declare that the research was conducted in the absence of any commercial or financial relationships that could be construed as a potential conflict of interest.
